# Practical Medicine

**Published:** 1876-01

**Authors:** 


### V. Practical Medicine.

1. *Pneumatometry.* Elsberg. (*N. Y. Med. Jour.*, Nov., 1875.)

Pneumatometry is the method of measuring the force of expiration and inspiration. For clinical purposes, the maximum force is measured. This is nearly constant for the same person, and the value of the method in diagnosis consists in observing the deviation from health of either inspiration or expiration, and the relation of the two. The instrument used for the purpose consists of an U shaped glass tube, partially filled with mercury, to one end of which is attached a rubber tube connected with a mouth-piece. As the patient inspires from or expires into this tube, the mercury rises or falls in the proximal arm of the glass tube—the amount being told by a scale fixed to the tube.

In health, the average of maximum inspiratory power for the male sex, is expressed by a column of mercury of 86 millimetres; of expiratory power, 110 millimetres. For the female sex, these quantities are expressed by 50 and 70 millimetres respectively. The expiratory power is thus seen to be normally greater than the inspiratory. This is due to the relations of the muscular power used in respiration, the elasticity of the lungs, and "the obstacles presented by the walls of the chest, its contents and their surroundings." In inspiration, "the muscular power forms the positive, and the elasticity of the lungs and the obstacles the negative, factors; while in expiration, all these three factors are positive, with only a relatively small negative one from expiratory obstacles."

Pulmonary emphysema may be detected in its incipient stage, "while yet all other methods of examination fail." The normal relation between the expiratory and inspiratory pressure is found to be inverted; the positive (expiratory) pressure is smaller than the negative (inspiratory). In the slighter forms of the disease, there is only a lessened expiratory pressure; in the more severe forms, the inspiratory power is lessened as well.

Asthma and bronchitis, especially chronic and recurring forms of the latter, are frequently accompanied by a condition of emphysema, and when this is the case, pneumatometry readily detects it. In phthisis, the pneumatometer shows an insufficiency in inspiratory pressure, while the expiratory, "for some time almost normal, is more slowly lessened, and remains larger than the inspiratory." Herein is a difference from emphysema and bronchitis, so great that it is of much value for differential diagnosis in doubtful cases, especially of incipient phthisis. In pneumonia and pleurisy, "similar pneumatometrical conditions obtain," as in phthisis pulmonalis.

2. *Frontal Headache.* BRUNTON. (*The Practitioner.*)

"The administration of a brisk purgative or small doses of Epsom salts thrice a day, is a most effectual remedy for frontal headache combined with constipation; but if the bowels are regular, the morbid processes on which it depends, seem to be checked, and the headache removed, even more effectually by nitro hydrochloric acid, or alkalis, given before meals. If the headache is immediately above the eyebrows, the acid is best; but if it is a little higher up, just where the hair begins, the alkalis appear to be more effectual. At the same time that the headache is removed, the feelings of sleepiness and weariness which frequently lead the patients to complain that they rise more tired than they lie down, generally disappear."

3. *Progressive Pernicious Anæmia, or Anæmatosis.* PEPPER. (*Am. Jour. Med. Sciences,* Oct., 1875.)

Prof. P. reports three cases of this disease.

Case I., was that of a single woman of 26 years, a stout dressmaker, with good family history, who had intense anæmia; irregular fever, uncontrolled by quinia; more or less œdema, gastric disturbances, palpitation, hæmic murmurs—"a humming roar audible over the whole skull," especially strong over the longitudinal and lateral sinuses"—profuse hæmorrhages from the gums, and, later, somnolence, coma and death. There was no albuminuria, no enlargement of spleen or lymphatic glands, and no evidence of organic disease of any organ.

Case II., was that of a man aged 57, who had through life had vigorous health. The disease was ushered in, after some months of somewhat impaired vigor, by a slight sunstroke; jaundice and rapid failure in strength and progressive anæmia followed. There were palpitation, nausea, vomiting, slight œdema, faintness on rising, hæmic murmurs, slight fever, slight emaciation, finally delirium and death. There was no leukemia or affection of the lymphatic glands. There was found slight enlargement of the spleen, and fatty degeneration of the heart, liver and kidneys.

Case III., was that of a man, an iron founder, slight and never vigorous, aged 50, who had had several times attacks of hepatic colic, with a chronic follicular catarrh of the intestinal canal, which had continued for a number of years. He had suffered for years from a chronic psoriasis of the legs, trunk and arms. The next morning, after a hard day's work, he felt weak; from this time there was progressive anæmia and debility, a tendency to syncope, transient œdema, hæmic murmurs, dyspnœa, irregular fever and somnolence. Later there was wandering delirium. There was found a large calculus in the gall-bladder, with suppuration of the sac; the solitary follicles of the intestines were enlarged; the heart, liver and kidneys were in a condition of fatty degeneration; the spleen was slightly swollen; the lymphatic glands were unaffected. The marrow of the bones (radius) was made almost wholly of "granular cells, round or nearly so, but varying in size from 1-3500 to 1-2000 of an inch. Many of these cells had a single distinct spherical nucleus; a few were very granular, and fewer contained a single drop of fat.

In all the cases there had been found a great deficiency of red corpuscles of the blood, but no increase of the white ones—the blood had, moreover, an appearance of disorganization. In the first case, death occurred in about a year from the beginning of the attack; in the second, in eleven months; and the third in three months.

The author, after a lengthy discussion of the cases and the disease, concludes that, (1) progressive pernicious anæmia is identical with the idiopathic anæmia of Addison, and is not a new disease; (2) it is in reality the medullary form of so-called pseudo-leukemia, (Hodgkin's disease); (3) the primary and essential lesion in this and analogous conditions appears to be an affection of the blood-making tissues—spleen, lymphatic glands, marrow of the bones—causing defective blood-making. A better name than any heretofore in use ought to be chosen for it, and he suggests *anæmatosis* (*α*, privative, and ʽαιματοσις, formation of blood); (4) the changes in the blood consist of great reduction of its mass, diminution of red without increase of white corpuscles; (5) the other lesions, fatty degenerations, etc., are secondary, and depend on the blood changes; (6) the symptoms are, in great part, explicable by the state of the blood and heart; (7) the disease, once established, is fatal; (8) the remedies affording most relief, are cod liver oil, arsenic and phosphorus; (9) transfusion is only capable of doing temporary good; and (10) to be safely employed, the amount of blood transfused should be small, and introduced slowly, and repeated at suitable intervals.

In the cases reported, all treatment was of no avail, including the transfusion, twice performed in the last case.

4. *Typhoid Fever, from Drinking Water.* Brown. (*Phil. Med. Times,* Nov. 13.)

In Mansfield, Pa., students of the Normal School began to have typhoid fever in Oct., 1874. By Dec., 53 had been attacked. There were treated in the school buildings 28 cases, 3 of which died; at their homes 25 cases, 5 of which died. The clinical history of the cases did not differ from that of ordinary, severe, typhoid fever. There was early diarrhœa in all the cases treated by Dr. B. (28), as also the rose rash in the second and third week. "All the patients vomited large quantities of bile during the period of high temperature."

The water supply of the school was from an artesian well, 140 feet deep. Near by was a surface-well, 20 feet deep; 40 feet from this, down the hill, was a large privy vault that had been used for 12 years. This vault was emptied by a sewer; a few feet below the vault, this sewer was entered by a drain extending from near the surface-well, to carry off surface-water from around the well. Many of the students preferred the water from the shallow well. A man working about the buildings who drank from this well, was attacked with the disease, although he resided at a distance. None of the hired help had the fever except the only person who drank habitually from the surface-well.

Prof. Latimer, of Rochester, found the water of the artesian well contained only "a small quantity of mineral matter," but that the water of the surface-well "literally swarmed with fungoid organisms, and also contained many animalculæ, besides much *débris* of both these classes of organisms in various stages of decomposition." Chemical tests gave strong evidence of sewage contamination. Dr. B. thinks the sewage from the vault had gradually backed up the well-drain, and finding its way through the soil, had contaminated the water which did the mischief.

5. *Three Cases of Dilatation of Lymphatic Radicles.* Dr. C. Hanfield Jones. (*Lancet*, Oct., 1875.)

The first case was that of a young man with grave disease of the kidneys, in which great quantities of albumen and some slender homogeneous and granular casts were voided with the urine. Both lower extremities were œdematous, the left most so, and there was for a time dullness over the lower right back. The abdomen "in both flanks" was markedly dull, the left being most affected, and being lessened on turning on the right side.

At the end of three months, the skin of the left thigh presented many "plexiformly arranged streaks" running upward. They soon increased in size so that they occupied a considerable part of the space of the surface of the thigh and buttock, but not passing above Poupart's ligament. Soon the same appearances, but in less degree, showed themselves on the opposite thigh. The man recovered.

The second case was in a man of 45 years, far gone in tubercular consumption, in whom just before death there appeared enlarged lymphatics similar to those of the first case, extending from the toes to the ankles.

The third case was in a young man with Bright's disease, who died from the affection at the end of eight weeks from the time of attack. Two weeks before death there appeared upon the surface of the lower abdomen and thighs many enlarged lymphatics converging toward Poupart's ligament. In a few days they became enormously distended with a reddish fluid.

In this case there was found, *post mortem*, a quantity of puriform fluid in the abdominal cavity; there was evidence of a slight peritonitis; the kidneys were very hyperæmic; the lymphatic glands of the mesentery were enlarged, but the glands near Poupart's ligament in the groin were not enlarged, nor were the receptaculum chyli or thoracic duct dilated.

The vessels enlarged in these cases, were not the ordinary subcutaneous, lymphatic vessels, for these run beneath the corium of the skin; the vessels in these three cases were situated *in* the corium or between it and the epidermis. They appeared "to be rather dilated, lymphatic radicles, than actual vessels."

Some of the fluid was drawn from the cavities in the first case, and it had all the appearance of lymph, a "clear alkaline fluid, devoid of corpuscles, slightly albuminous, sp. gr. 1009." The fluid must have been in rapid motion, as from a single needle puncture the flow was so great that two clean sheets were saturated, and 1½ oz. of the material was collected.

Dr. J. is unable to fully account for the appearances described, but thinks the most rational theory of explanation is that, as there was in all the cases more or less venous obstruction, "as the return of blood was barred, there was no other channel for the fluid effused from the capillaries to take but the lymphatics."

